# A randomized, double-blind clinical trial of a herbal formulation (GlycaCare-II) for the management of type 2 diabetes in comparison with metformin

**DOI:** 10.1186/s13098-021-00746-0

**Published:** 2021-11-17

**Authors:** Muhammed Majeed, Anju Majeed, Kalyanam Nagabhusahnam, Lakshmi Mundkur, Shaji Paulose

**Affiliations:** 1 Sami-Sabinsa Group Limited, 19/1 & 19/2, I Main, II Phase, Peenya Industrial Area, Bangalore, 560 058 Karnataka India; 2 Sabinsa Corporation, 20 Lake Drive, East Windsor, NJ 08520 USA

**Keywords:** GlycaCare-II, Metformin, Glycosylated hemoglobin, Fasting blood sugar and postprandial blood sugar, Prediabetic, Newly diagnosed diabetic and type 2 diabetes mellitus

## Abstract

**Background:**

Type 2 diabetes mellitus (T2DM) is a major public health concern with growing prevalence with multiple debilitating complications. GlycaCare-II is a proprietary herbal formulation supplement for T2DM containing extracts of *Cinnamomum cassia, Momordica charantia, Pterocarpus marsupium, Gymnema sylvestre, Salacia reticulata, Eugenia jambolana*, and a bioavailability enhancer piperine from *Piper nigrum*.

**Objective:**

The antihyperglycemic potential of GlycaCare-II was compared against metformin in a double-blind study.

**Design:**

It was a randomized, two-arm design on prediabetic (N = 29; 12 in metformin and 17 in GlycaCare-II arm, respectively) and newly diagnosed diabetic (N = 40; 16 in metformin and 24 in GlycaCare-II) patients for 120 days.

**Outcome measures:**

Changes in diabetic panel glycosylated hemoglobin (HbA1c), fasting blood sugar (FBS), and postprandial blood sugar (PBS) were the primary endpoints. Lipid profile, liver profile, thyroid-stimulating hormone, bilirubin and creatinine were the secondary endpoints.

**Result:**

Twice a day treatment for 120 days with GlycaCare-II led to a statistically significant change in HbA1c (p < 0.001), FBS (p < 0.001), PBS (p < 0.001) on both prediabetic and newly diagnosed diabetic patients. GlycaCare-II showed a similar potential as metformin in the treatment of T2DM. In the prediabetic group, both GlycaCare-II and metformin were comparable for all the hyperglycemic index parameters. In the case of newly diagnosed diabetic patients, GlycaCare-II showed a significantly better reduction for PBS (p = 0.026) as compared to metformin, while all other parameters in the diabetic panel were comparable. No adverse events were reported throughout the trial period.

**Conclusion:**

These results suggest that GlycaCare-II is effective in managing T2DM in both newly diagnosed diabetic and prediabetic patients.

## Introduction

Type 2 diabetes mellitus (T2DM) is a persistent hyperglycemic disorder, wherein blood glucose levels are above the normal values. Further, it is also characterized by an increase in oxidative stress [1, 2]. T2DM is a major public health concern with multiple debilitating complications. Despite considerable improvement in medical sciences, diabetes mellitus is still an incurable disease rapidly increasing in all age groups [3]. The International Diabetes Federation estimated that the global diabetes prevalence in 2019 is 463 million people, rising to 578 million by 2030 and 700 million by 2045 [4]. Hyperglycemia causes both macrovascular (coronary artery disease, peripheral arterial disease, and stroke) and microvascular complications (diabetic nephropathy, neuropathy, and retinopathy) [5]. The overall glycemic burden over time as measured by glycosylated hemoglobin (Hb1Ac) determines the risk for microvascular complications [6].

T2DM is treated by numerous drugs to increase glucose metabolism and insulin secretion. Biguanide, Sulphonylureas, Alpha-glucosidase inhibitors, Thiazolidinediones, and Gliptins are the commonly prescribed medication for T2DM [7]. Most of these drugs reduce circulating glucose levels and HbA1c to a similar extent but differ in their safety and pathophysiological effects [8]. Metformin is accepted as the first-line therapy for T2DM. It has an extensive safety margin, decreases hepatic glucose production, and mildly affects peripheral resistance [9]. Lactic acidosis, drowsiness, muscle pain gastrointestinal problems are a few side effects associated with metformin, while some people experience B-12 deficiency [9]. Sulfonylureas help increase insulin secretion and may also increase the responsiveness of pancreatic β-cells to glucose. They are well tolerated, although hypoglycemia and weight gain are the most common side effects. Further, their long-term durability effect is inferior to metformin [10]. The PPAR-γ agonists maintain long-term control of blood glucose levels by reducing insulin resistance and improving β-cell function. Bodyweight gain and fluid retention are the major adverse effect of this class of antidiabetic drugs [11] The glucose-dependent insulinotropic polypeptide (GIP) and GLP-1 are peptide hormones (incretins) secreted in the small intestine, which activate insulin secretion in healthy individuals. Incretin mimetics and dipeptidyl peptidase-4 (DPP-4) inhibitors have a positive effect on sustained improvement in glycemic control and on weight gain [12]. The safety of DPP-4 or GLP-1 therapy over time is not yet clear as thyroid cancer and pancreatitis have been reported [8]. Although initial response to drugs like metformin may be good, oral hypoglycemic drugs lose their effectiveness in a significant percentage of patients [13].

In recent decades, there has been a collective determination for pursuing alternative medicine for the treatment of T2DM from natural or herbal sources [14]. Other factors such as patient compliance have led the way in trying out alternative, complementary medicine. Nutritional therapy is gaining importance in preventing, managing, and slowing the rate of development of diabetes complications. It is, therefore, important at all levels of diabetes prevention [15].

Medicinal herbs from India and China have been widely used for more than 2000 years to treat type 2 diabetes mellitus [16]. The mechanism of action of the herbal medicines involves modifying glycemic metabolism, reducing cholesterol levels, and facilitating insulin secretion [17].

GlycaCare-II® is the formulation containing *Cinnamomum cassia*, *Momordica charantia, Pterocarpus marsupium*, *Gymnema sylvestre*, *Salacia reticulata*, *Eugenia jambolana* and piperine from *Piper nigrum*. The cinnamaldehyde in *C. cassia* sensitizes the body to insulin by enhancing insulin-stimulated tyrosine phosphorylation [18], while the cinnamon polyphenols display insulin-like activity [19]. *M. charantia* has molecules like charantin, vicine, and polypeptide-p(insulin-like hypoglycemic protein), which possess an antihyperglycemic effect with a mechanism similar to insulin [20]. Thus, some of the proposed mechanism of *M*. *charantia* in T2DM includes insulin-like effects and reduction in glucose absorption [21].

*P. marsupium* extract has been documented to help in protection against oxidative stress in diabetes [22]. Pterostilbene, present in the extract, normalizes serum insulin levels and reduces oxidative stress in diabetic rats [23]. The C-glycosides present in *P. marsupium* was found to increase glucose uptake by skeletal muscles and could be the active constituent responsible for antihyperglycemic activity [24].

Gymnemic acid from *Gymnema sylvestre* is a mixture of at least 23 different saponins with a similar atomic arrangement to glucose. It acts as an antihyperglycemic agent by filling the receptor location, preventing sugar molecules' absorption by the intestine [25]. Salacinol and Mangiferin from *Salacia reticulata* inhibit the alpha-glucosidase enzyme, thus decreasing the plasma glucose level [26, 27].

The bark of *Eugenia jambolana* is rich in several bioactive compounds [28–30]. Its fruits contain raffinose which has hypoglycemic activities [31–35]. The blood-glucose-lowering effect of *Eugenia jambolana* may be due to increased secretion of insulin from the pancreas or by inhibition of insulin degradation [36].

Piperine enhances the absorption of nutrients through epithelial cell modification and promotes permeability [37]. Piperine, through its multifaceted effect on bioavailability has an indirect impact in the treatment of T2DM [38, 39].

Although various herbal products are in use for T2DM, only a few products have been compared with metformin, and even in the comparison, the outcome of antihyperglycemic activity was lower than that of metformin [40]. Hence, the purpose of this study was to evaluate and compare the efficacy and safety of GlycaCare-II against metformin for the management of T2DM in prediabetic and newly diagnosed patients.

## Materials and methods

### Test product

GlycaCare-II® tablets (522.5 mg) was manufactured and provided by Sami-Sabinsa Group Limited (erstwhile Sami Labs Limited), India. GlycaCare-II® contains the following ingredients:

**Table:** 

Sl.no	Ingredients	Qty/mg	Percentage (%) of Actives
1	Cinnamon Extract	150	20% polyphenols
2	*Momordica charantia* Extract	150	0.5% Charantin
3	Pterocarpus Extract (Water-soluble)	150	5% C-glycosides
4	*Gymnema sylvestre* Extract	30	25% gymnemic acids
5	*Salacia reticulata* extract	20	1% Mangiferin
6	*Eugenia jambolana* extract	20	< 15% Tannins
7	Piperine(Bioperine)	2.5	95% Piperine

Cinnamon (*Cinnamomum cassia*) bark, Gymnema (*Gymnema sylvestre*) leaves, deseeded *(Momordica charantia*) fruits, Dried fruits of Jamun(*Eugenia jambolana*), and Dried Salacia bark (*Salacia reticulata*) were powdered and extracted with methanolic water at refluxed condition. The extract was concentrated to remove methanol, dissolved in water, and spray dried. Dried Pterocarpus wood (*Pterocarpus marsupium*) was extracted with water.

Metformin (GLYCIRITE) tablets (500 mg) was manufactured by Tusker Pharma India Pvt. Ltd, India.

### Study design

The present study was a prospective, randomized, double-blind, active-controlled clinical trial. Its primary objective was to evaluate the efficacy and safety of GlycaCare-II as monotherapy in Type 2 diabetes mellitus patients compared to metformin. Enrolled patients were initially segregated into prediabetic patients and newly diagnosed diabetic patients. The patients were randomly allocated into two treatment groups to prevent treatment bias. The patients and investigators were blinded to the treatment allocation. Out of 70 subjects screened, sixty-nine subjects enrolled in the study. All the enrolled patients were randomized to two treatment groups: Treatment 1: GlycaCare-II (522.5 mg) as active or Treatment 2: Metformin (500 mg) as the comparator. During the treatment phase, 29 prediabetic patients and 40 newly diagnosed diabetic patients with Type 2 Diabetes mellitus were randomized to receive either GlycaCare-II or metformin under each arm for a period of 120 days ± 3 days. Investigational Product (IP) was administered orally twice daily, morning and night, 20 min before food. All the participants signed informed consent before the beginning of the study after careful detailing regarding the purpose, procedure, and potential risks and benefits of the study.

### Study population

Subjects within the age group of 30–65 years, having the ability to comply with the study protocol and willing to give written consent, were included in the study. Prediabetes was classified as per American diabetes association criteria HbA1c 5.7–6.4% and FBS between 100 mg/dL to 125 mg/dL. Newly diagnosed diabetes patients had an HbA1c value of 6.5–7.5% and FBS > 125 mg/dL [41]. Pregnant and lactating women, patients with a history of acute or chronic illness, type I diabetes, hypo-, and hyperthyroidism were excluded from the study. Also, subjects with hyperlipidemia, history of severe hepatic dysfunction or renal dysfunction, uncontrolled pulmonary dysfunction, and poorly controlled hypertension were excluded from the study. Any patient did not use concomitant medications during the course of the trial.

**Fig. 1:**
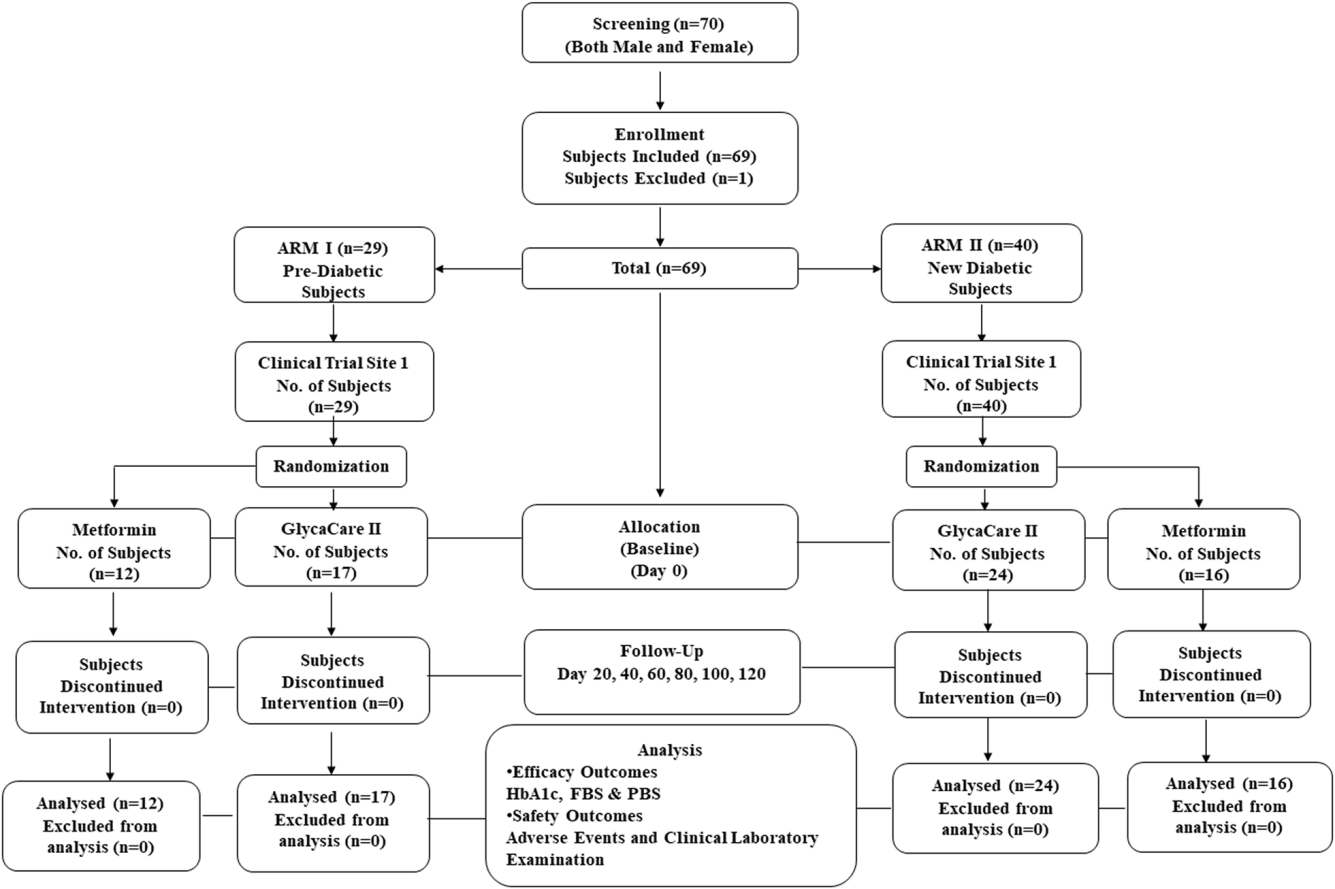
Consort flow diagram

Details of the subject’s disposition are presented in the consort flow chart (Fig. 1).

### Ethics and informed consent

The study was planned at two centers; however, executed at only one site, Levin Diabetes Specialty Hospital, Madurai. The institutional ethics committee of both the hospitals approved it. However, the trial activity was terminated at the initial phase of the study at Pristine Hospital & Research Centre (P) Ltd due to non-compliance. The study was conducted on 69 subjects instead of the proposed 140 subjects. A protocol deviation pertaining to the change in the number of subjects was filed to the ethics committee, and the changes were duly updated in the Clinical Trial Registry of India (CTRI) with registration number CTRI/2018/02/012085 on February 22, 2018, retrospectively. Written Informed Consent was taken from all the subjects before enrolling in the study.

### Data collection, compliance, and protocol deviation

This study was conducted in accordance with applicable regulations, GCP, and Standard Operating Procedures. Study monitor(s) from ClinWorld monitored the study process and data collection through periodical site visits. The monitor retrieved the CRFs (Case Report Form) upon satisfactory resolution of all the queries. Investigational Product (IP) compliance was maintained for both active tablets GlycaCare-II and comparator Glycirite tablets. IP compliances were assessed through CRF. There were no deviations observed regarding IP compliance, during the treatment.

### Study outcome

Change in diabetic panel (Glycosylated hemoglobin (HbA1c), fasting blood sugar (FBS), and postprandial blood sugar (PBS)) is the primary endpoint. In case of secondary endpoints, adverse events and change in the biochemical parameters viz lipid profile (Total cholesterol (TC), triglycerides (TG), low-density lipoprotein (LDL), high-density lipoprotein (HDL), very low-density lipoprotein (VLDL)), liver profile (aspartate transaminase (AST), alanine transaminase (ALT)) and renal profile (Serum creatinine) were performed. All routine clinical chemistry parameters were analyzed using Erba Chem 5® Plus V2 (ERBA Diagnostics Mannheim GmbH Mallaustrasse 69–73 68,219 Mannheim, Germany). Hematology was analyzed using 6 part cell analyzer, SYSMEX, XN-150, (Mumbai, 400 078, Maharashtra, India). HbA1c was analyzed using a D-10 analyzer, BIO-RAD Laboratories (Hercules, CA, USA).

Efficacy and safety parameters were assessed during the patients' visits to the site. Physical examination, demographics (height, weight, body mass index (BMI)), vital signs were assessed at each visit of the subjects. Clinical efficacy parameter HbA1c was assessed at the screening visit (day − 3) and final visit (day 120 ± 3). FBS and PBS were assessed at the screening visit (day − 3), visit 3 (day 20 ± 3), visit 4 (day 40 ± 3), visit 5 (day 60 ± 3), visit 6 (day 80 ± 3), visit 7 (day 100 ± 3) and final visit (day 120 ± 3). All the biochemical parameters viz thyroid profile (thyroid-stimulating hormone-TSH), lipid profile, liver profile, and renal profile were assessed at the screening visit (day − 3) and final visit (day 120 ± 3).

### Statistical analysis

All patients in the study with relevant safety and efficacy data were considered for the analysis. Efficacy and safety endpoints were analyzed for the relevant study population. A descriptive analysis of demographic characteristics was performed. Mean, and the standard deviation was derived for numeric and categorical parameters. Vital signs at each visit were also analyzed descriptively. Both primary and secondary efficacy outcomes were analyzed descriptively.

For normally distributed data, parametric tests have been applied, and results on continuous measurements were presented as mean ± SD, and results on categorical measurements were presented in percentage (%). A statistical significance level of ≤ 5% was considered significant. Fasting and postprandial glucose levels have been evaluated using repeated-measures ANOVA. HbA1c levels at the screening visit (visit 1) and at the end of the treatment were evaluated using student's ―paired t-test.

As part of safety outcomes, adverse events, concomitant medications, and clinical laboratory data were assessed. Clinical laboratory outcomes were assessed descriptively. Mean and standard deviation were derived from the data. The p-value for each efficacy parameter and for individual laboratory parameters was calculated using the Wilcoxon test.

## Results

### Demographics and other baseline characteristics

A total number of 69 subjects were enrolled and completed the study with an average age range of 48–52.9 years. The demographic parameters were comparable between the metformin and GlycaCare-II treatments at baseline. The other demographic parameters are as shown in Table 1.

**Table 1:** Demographics and baseline Characteristics

Parameters	Prediabetics				Newly diagnosed diabetics			Overall average
	GlycaCare-II (N = 17)		Metformin (N = 12)		GlycaCare-II (N = 24)		Metformin (N = 16)	
Average Age (Yrs)	49.6		48		51.3		52.9	50.45
Average height (cm)	164.4		154.5		162.3		160.2	160.35
Average weight (kg)	72.2		64.3		65.6		65.8	66.98
BMI (kg/m^2^)	26.87		28.31		25.02		25.57	26.44
Systolic Blood Pressure (mmHg)	123.88		120.42		124.29		131.63	125.06
Diastolic Blood Pressure (mmHg)	79.47		77.67		79.29		81.63	79.51
Pulse rate (beats/min)	83.88		81.50		85.08		84.25	83.67

						Overall
Gender	Male	9	3	17	4	33
	Female	8	9	7	12	36
Non-smokers		17	12	24	16	69
Non-alcoholics		17	12	24	16	69

**Fig. 2:**
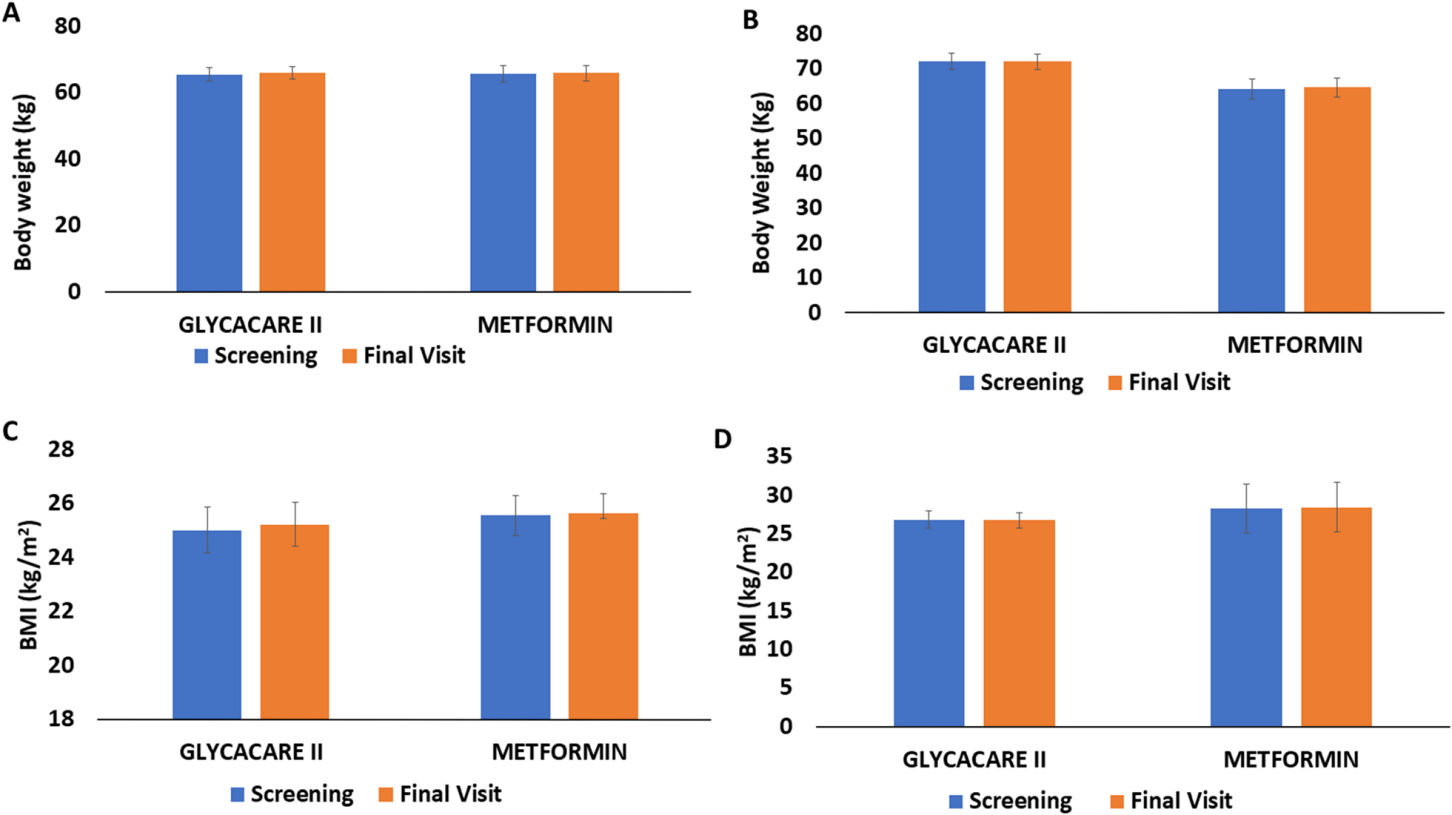
Bodyweight and BMI comparison from screening to final visit. **A** Body weight change in newly diagnosed diabetic group **B** Bodyweight change in prediabetic group **C** BMI change in newly diagnosed diabetic group **D** BMI change in prediabetic group; Values are expressed as Mean ± SE

Bodyweight and BMI were compared between screening and final visit (Fig. 2A–D)

*cm *centimeters, *kg* kilogram, *BMI* Body Mass Index

### Efficacy analysis of GlycaCare-II and metformin

#### Newly diagnosed diabetic patients

##### Effect on HbA1c level

**Fig. 3:**
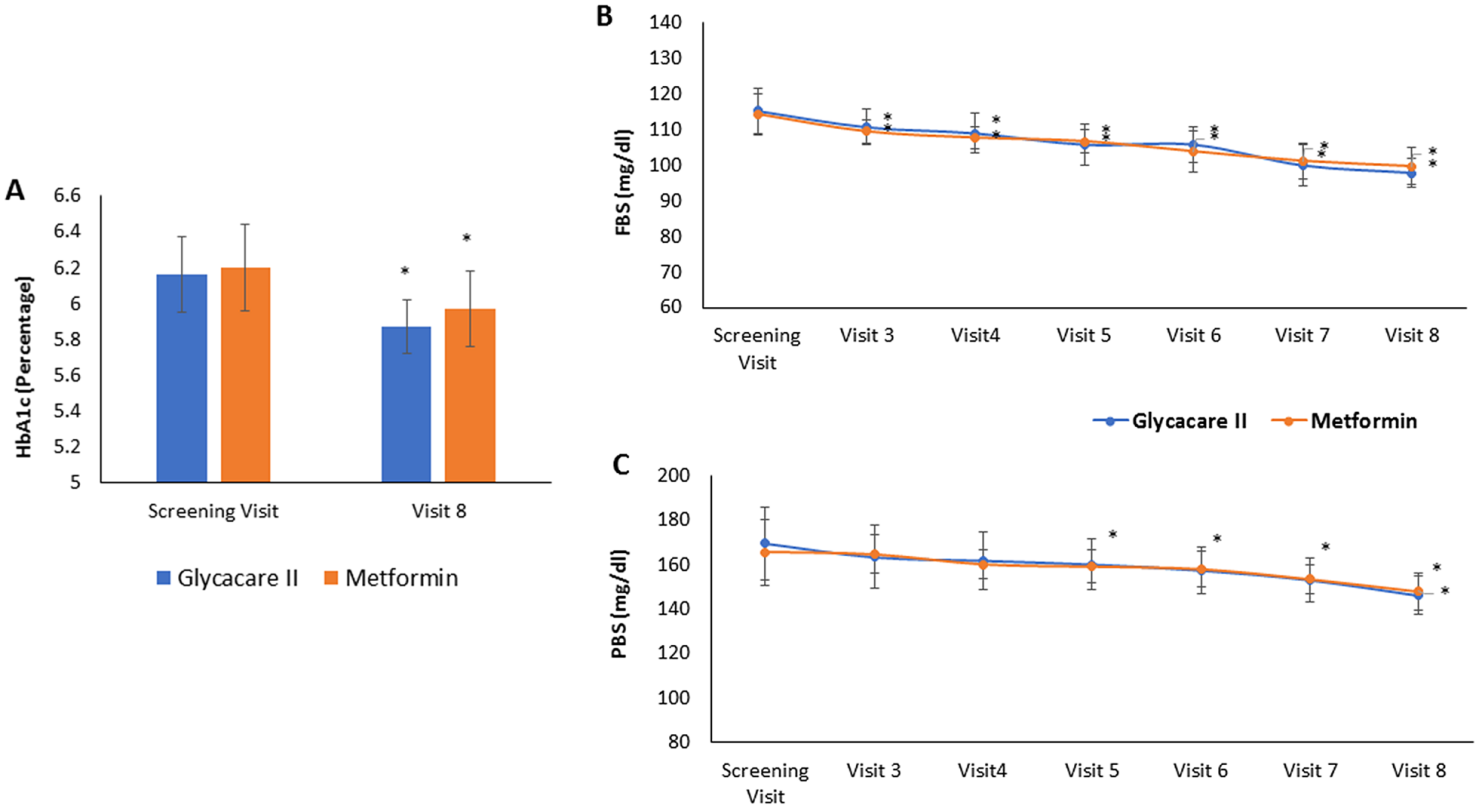
Efficacy parameter for GlycaCare-II and Metformin in newly diagnosed diabetic patients, FBS and PBS was measured at the screening visit (visit 1), visit 3, visit 4, visit 5, visit 6, visit 7 and visit 8and HbA1c was measured at screening and final visit (visit 8). **A** HbA1c level in %, **B** Fasting blood sugar level in mg/dL, **C** Postprandial blood sugar level in mg/dL. (Values are expressed as Mean ± SD, *p < *0.05* in comparison to screening visit by repeated measure ANOVA)

One of the primary efficacy parameters recorded for this clinical study was a change in the level of HbA1c from screening visit to final visit. In the case of newly diagnosed diabetic patients, the HbA1c level was 6.99 ± 0.32% for GlycaCare-II and 6.99 ± 0.38% for the metformin group at the screening visit. In the final visit, the HbA1c level reduced to 6.52 ± 0.19% (p < 0.001) for GlycaCare-II and 6.53 ± 0.26% (p = 0.004) for metformin. The mean changes in HbA1c were not significantly different in metformin & GlycaCare-II groups, suggesting equivalent efficacy (Fig. 3A).

##### Effect on fasting blood sugar

FBS level recorded a reduction of 11% for GlycaCare-II, and a similar improvement in the FBS level was observed in the metformin arm with a 10% reduction at visit 8 (Fig. 3B).

##### Effect on postprandial blood sugar

A significant change in the PBS level at the final visit as compared to the screening visit was observed in both GlycaCare-II (p < 0.0001) and metformin (p = 0.0005) groups in the newly diagnosed diabetes patients. Total change in the PBS level was significant between the treatment groups (p = 0.0268). In the case of PBS level, GlycaCare-II displayed a significant therapeutic response compared to metformin with the absolute mean change of 32.75 mg/dl as against 21.06 mg/dL of metformin group. Additionally, a significant change was observed in the PBS value from visit three onwards within the group for GlycaCare-II and metformin. Further, these changes were incremental across the time points for both groups (Fig. 3C).

#### Prediabetic patients

##### Effect on HbA1c level

**Fig. 4:**
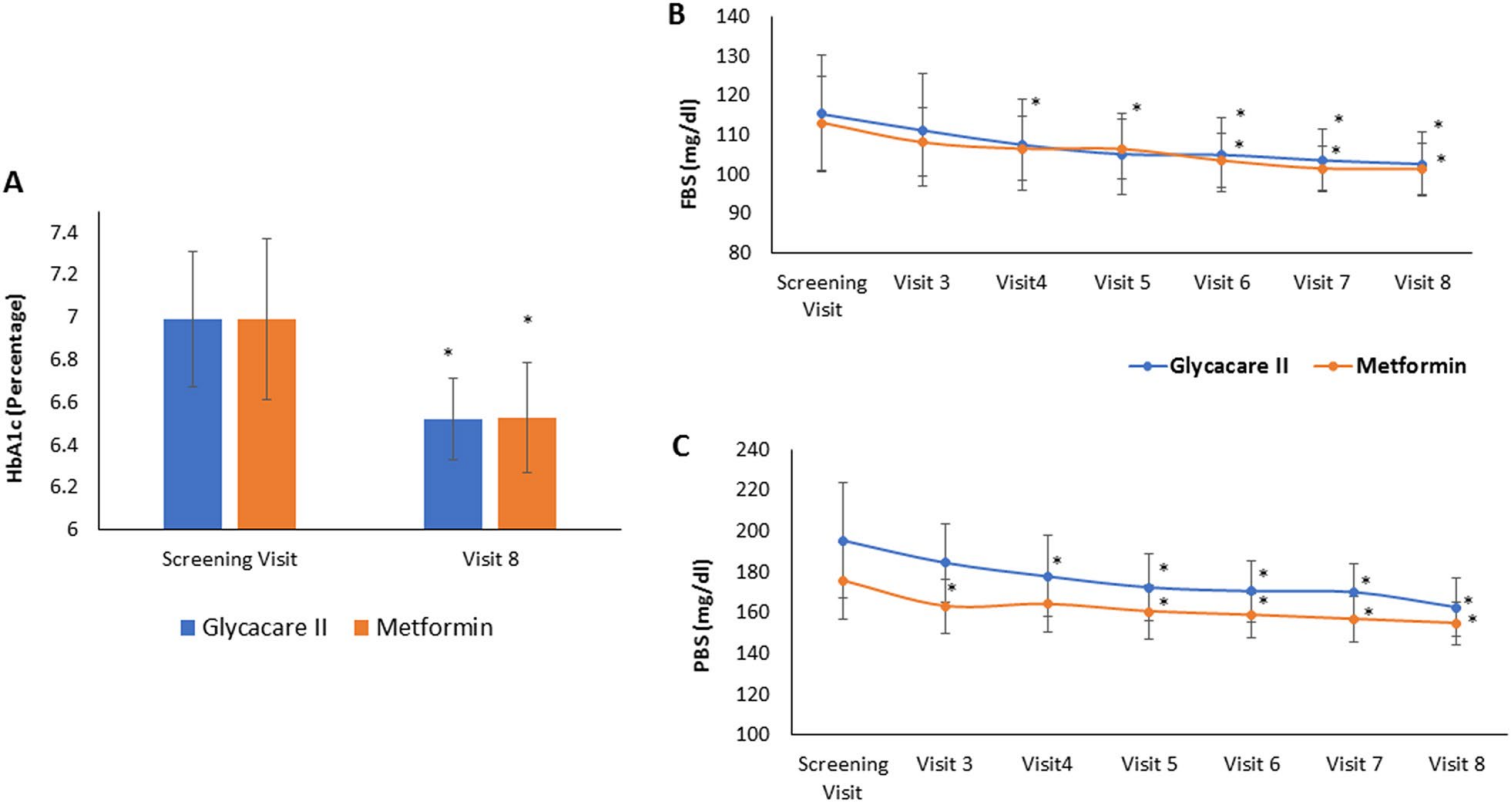
Efficacy parameter for GlycaCare-II and metformin in prediabetics arm, FBS and PBS was measured at the screening visit (visit 1), visit 3, visit 4, visit 5, visit 6, visit 7 and visit 8, HbA1c was measured at screening and final visit (visit 8). **A** HbA1c level in %, **B** Fasting blood sugar level in mg/dL, **C** Postprandial blood sugar level in mg/dL. (Values are expressed as Mean ± SD, *p < 0.05 in comparison to screening visit by repeated measure ANOVA)

The mean HbA1c changed from 6.16 ± 0.21 to 5.87 ± 0.15% (p < 0.001) in GlycaCare-II group and 6.2 ± 0.24 to 5.97 ± 0.21% (p = 0.02) in metformin group, from screening visit to final visit. The mean changes in HbA1c were not significantly different in metformin & GlycaCare-II groups, suggesting equivalent efficacy (Fig. 4A).

##### Effect on fasting blood sugar

A significant change was observed in the FBS level from Day 20 onwards for both GlycaCare-II and metformin group, and the changes were incremental across the time points with resultant 15% and 11% reduction at 120 days, respectively in the pre-diabetics group (Fig. 4B).

##### Effect on postprandial blood sugar

In the prediabetes patients, the PBS level visit-wise for both GlycaCare-II and metformin showed steady augmentation over a period. The PBS level for GlycaCare-II at the screening visit was 169.59 ± 16.35 mg/dl and for the final visit 146 ± 8.66 mg/dl (p < 0.0001). For metformin it was 165.67 ± 14.89 mg/dl at screening visit (day 3) and 148 ± 8.31 mg/dl for final visit (day-120 ± 3) (p = 0.0016). Additionally, a significant change within the group was observed in the PBS level from visit five onwards for GlycaCare-II, and the changes were incremental across the time points. However, a significant change was observed for the metformin group from visit 6, with the incremental difference across the time points (Fig. 4C).

### Safety evaluation

There were no adverse events or serious adverse events observed, and none of the patients consumed any concomitant medications during the study in both prediabetic and newly diagnosed diabetic arms.

### Clinical laboratory evaluation

#### Lipid levels

The reduction in TC, TG, LDL, VLDL, and the increase in HDL was highly significant in the GlycaCare-II treated subjects. Metformin did not show a significant reduction in TG and VLDL. The positive effect on lowering lipids was better with GlycaCare-II compared to metformin. This effect was not observed in prediabetic subjects (Table 2).

**Table 2:** Lipid profile for GlycaCare-II and Metformin

Group	Product	Prediabetic			Newly diagnosed diabetic		
Day		0	120	P-value	0	120	P-value
Parameter		Mean ± SD	Mean ± SD		Mean ± SD	Mean ± SD	
TC (mg/dl)	GlycaCare-II	194.29 ± 22.26	180.88 ± 20.35	0.0761	208.96 ± 25.18	187.04 ± 16.27	0.0008↓*
	Metformin	184.17 ± 14.78	179.00 ± 15.71	0.4153	198.31 ± 12.84	185.69 ± 15.10	0.0162↓*
TG (mg/dl)	GlycaCare-II	162.00 ± 21.98	157.00 ± 18.88	0.4819	172.13 ± 18.44	157.13 ± 14.61	0.0031↓*
	Metformin	151.50 ± 28.79	152.42 ± 13.60	0.9212	162.88 ± 14.87	158.31 ± 18.82	0.4519
HDL (mg/dl)	GlycaCare-II	38.82 ± 4.45	36.29 ± 3.84	0.0855	41.75 ± 4.87	37.46 ± 3.30	0.0008↓*
	Metformin	39.42 ± 6.84	35.17 ± 2.37	0.0542↓	40.00 ± 3.14	37.257 ± 3.47	0.0255↓*
LDL (mg/dl)	GlycaCare-II	120.41 ± 19.92	113.35 ± 13.29	0.2330	132.21 ± 17.81	118.13 ± 11.10	0.0019↓*
	Metformin	115.33 ± 10.06	110.92 ± 14.19	0.3893	125.88 ± 13.88	114.19 ± 12.29	0.0172↓*
VLDL (mg/dl)	GlycaCare-II	32.47 ± 4.56	31.24 ± 3.73	0.3957	34.58 ± 3.69	31.46 ± 2.81	0.0019↓*
	Metformin	30.42 ± 5.87	30.17 ± 3.97	0.9038	32.99 ± 3.06	31.63 ± 3.91	0.2819

*SD* Standard deviation, *TC* total cholesterol, *TG* TriGlycerides, *HDL* high-density lipoproteins, *LDL* low-density lipoproteins, *VLDL* very low-density lipoproteins, ↑* = Statistically significant increase (p < 0.05), ↓* = Statistically significant decrease (p < 0.05), No. of Subjects (Prediabetics: GlycaCare-II–17 & Metformin–12, Newly diagnosed diabetic: GlycaCare-II-24 & Metformin-16)

### Safety parameters

#### Newly diagnosed diabetic arm

Bilirubin, and creatinine, changed significantly in metformin group in comparison to baseline. It was also noted that the total bilirubin increased significantly in the patients treated with metformin at the end of the study in comparison to the GlycaCare-II group. The vital parameters did not show any significant changes across the study period in both arms. Nevertheless, the respiratory rate of the metformin group was significantly decreased at the end of the study. These results reiterate the safety of GlycaCare-II for human consumption in newly diagnosed diabetic patients (Table 3).

**Table 3:** Safety profile of GlycaCare-II and Metformin

Group	Product	Prediabetic			Newly diagnosed diabetic		
Day		0	120	P-value	0	120	P-value
Parameter		Mean ± SD	Mean ± SD		Mean ± SD	Mean ± SD	
TSH (µIU/ml)	GlycaCare-II	2.72 ± 0.59	2.55 ± 0.60	0.4110	3.08 ± 0.65	3.01 ± 0.66	0.7129
	Metformin	2.39 ± 0.94	2.70 ± 0.98	0.4375	3.04 ± 0.71	2.94 ± 0.71	0.6932
Total bilirubin (mg/dl)	GlycaCare-II	0.86 ± 0.14	0.85 ± 0.14	0.8364	0.89 ± 0.14	0.86 ± 0.12	0.4295
	Metformin	0.82 ± 0.13	0.78 ± 0.13	0.4590	0.71 ± 0.13	0.80 ± 0.06	0.0175↑*
AST (IU/L)	GlycaCare-II	27.47 ± 7.79	28.00 ± 6.48	0.8306	28.21 ± 7.05	28.00 ± 6.85	0.9171
	Metformin	24.58 ± 7.32	25.17 ± 5.91	0.8300	21.94 ± 8.34	22.94 ± 5.64	0.6940
ALT (IU/L)	GlycaCare-II	24.41 ± 3.69	22.94 ± 3.68	0.2534	26.83 ± 4.68	26.17 ± 6.18	0.6786
	Metformin	25.00 ± 4.02	26.25 ± 3.39	0.4191	25.19 ± 5.65	25.63 ± 5.06	0.8181
Total creatinine (mg/dl)	GlycaCare-II	0.86 ± 0.11	0.85 ± 0.11	0.7927	0.88 ± 0.13	0.87 ± 0.13	0.7911
	Metformin	0.91 ± 0.10	0.88 ± 0.21	0.6845	0.80 ± 0.21	1.07 ± 0.29	0.0052↑*

*SD* Standard deviation, *TSH* Thyroid Stimulating Hormone, *AST* Aspartate Aminotransferase, *ALT* Alanine Transaminase, ↑* = Statistically significant increase (p < 0.05), ↓* = Statistically significant decrease (p < 0.05), No. of Subjects (Prediabetics: GlycaCare-II–17 & Metformin—12, Newly diagnosed diabetic: GlycaCare-II-24 & Metformin-16)

#### Prediabetic arm

Clinical laboratory evaluation was carried out on the study participants during screening and final visit. The biochemical parameters during the screening and the final visit, showed no significant change across time. The vital parameters did not show any significant changes across the study period in both the arms. These results reiterate the safety of GlycaCare II and metformin for human consumption in prediabetic patients (Table 3).

## Discussion

In the present study, we observed that a herbal formulation (GlycaCare-II), containing natural extracts of *Cinnamomum cassia*, *Momordica charantia, Pterocarpus marsupium, Gymnema sylvestre, Salacia reticulata, Eugenia jambolana* with a small quantity of piperine as bioavailability enhancer was comparable to metformin in reducing hyperglycemia and HbA1c levels on both prediabetic and newly diagnosed diabetic patients.

Compared with the baseline data, significant improvement in all the primary biochemical indices of hyperglycemia like HbA1c, FBS, and PBS was observed in all the subgroups after four months of treatment. The GlycaCare-II formulation was safe with no changes in blood biochemical parameters, and no adverse effects were reported during the four months of treatment. In subjects treated with metformin, a significant increase in creatinine and bilirubin levels was observed in newly diagnosed diabetic patients. Although the number of subjects was low, this trend cannot be ignored and warrants a larger cohort study. Few earlier studies on chronic therapy of metformin reported a significant change in creatinine [42, 43].

GlycaCare-II exhibited a significant change in PBS level compared to metformin at the end of the study, especially in the newly diagnosed diabetic patients. Further, GlycaCare-II showed statistically significant improvement in lipid levels suggesting its benefit in controlling dyslipidemia. These inferences are in line with other studies, wherein individual components of this formulation were efficacious in bringing down metabolic index [18, 21, 40, 44–46]. The treatment of GlycaCare-II was devoid of any adverse events, and the outcome of the analysis of the laboratory parameters suggests that GlycaCare-II is safe for diabetic patients.

Optimal treatment of type 2 diabetes mellitus requires a comprehensive and concerted approach. The management of the condition focuses on nutrition, exercise, and pharmacologic therapies to reduce the complications associated with hyperglycemia. In prediabetic and new-onset diabetes patients, nutrition therapy is of utmost importance to prevent further deterioration of the condition [47]. The American diabetic association recommends HbA1c with a cut-point ≥ 6.5% for diagnosing diabetes as an alternative to fasting plasma glucose as it provides a reliable measure of chronic glycemia and correlates well with the risk of long-term diabetes complications [48]. Further, HbA1c is also a good predictor of lipid profile, providing additional benefits of identifying cardiovascular risk among diabetes patients [49]. GlycaCare-II was comparable to metformin therapy in reducing the HbA1c levels in both prediabetic and newly diagnosed diabetic patients. In addition, GlycaCare-II was highly effective in reducing lipid levels in newly diagnosed diabetic patients, which was better than the effect of metformin. These results suggest that GlycaCare-II can be a beneficial supplement for diabetic patients with dyslipidemia, which requires further elaboration. Although the lipid levels decreased in prediabetic patients also, it was not significant, probably because of the overall lower levels in these groups.

Postprandial hyperglycemia is also one of the earliest abnormalities of glucose homeostasis, and it has been suggested that lowering PPS may decrease the risk of hypoglycemia and weight gain [50]. GlycaCare-II was significantly better than metformin in reducing PPS in newly diagnosed patients, and the effect was seen at an earlier time point in prediabetic patients. These observations also suggest that GlycaCare-II may be used to successfully reduce the risk of developing diabetes in prediabetics and reduce progression in diabetic patients.

One limitation of the study was the inability to carry out the multicenter trial as per the original protocol due to non—compliance and subsequent termination of the site, resulting in smaller subgroups. We restricted the outcome parameters to only the glycemic profile as this was the first clinical study with GlycaCare-II. This can be considered a study limitation, as evaluation of insulin levels and oxidative parameters would have extended the benefits afforded by the herbal formulation.

## Conclusion

The findings in this randomized clinical study demonstrate the potential of GlycaCare-II as an alternative safe medication in the treatment of T2DM. It was also evident that GlycaCare-II possesses a similar therapeutic response as compared to metformin. Future studies in a larger cohort may help in positioning the polyherbal formulation as an alternative to the standard treatment for type 2 diabetes. We have shown that GlycaCare-II, with its steady influence on reducing the hyperglycemic index, is comparable with metformin. GlycaCare-II also appeared to have a better effect on the changes in the lipid parameters. However, the intrinsic cumulative mechanisms of its action must be further established through a comprehensive trial involving an increase in the number of subjects.

## Data Availability

All the data generated are within the manuscript.

## Abbreviations

AST: Aspartate transaminase
ALT: Alanine transaminase
BMI: Body Mass Index
CTRI: Clinical Trial Registry of India
FBS: Fasting blood sugar
FDA: Food and Drug Administration
HbA1C: Glycosylated hemoglobin
HDL: High-density lipoprotein
IP: Investigational Product
LDL: Low density lipoprotein
PBS: Postprandial blood sugar
T2DM: Type 2 diabetes mellitus
TC: Total cholesterol
TG: Triglycerides
TSH: Thyroid Stimulating Hormone
VLDL: Very low-density lipoprotein
